# The CONSTANCES Cohort Biobank: An Open Tool for Research in Epidemiology and Prevention of Diseases

**DOI:** 10.3389/fpubh.2020.605133

**Published:** 2020-12-10

**Authors:** J. Henny, R. Nadif, S. Le Got, S. Lemonnier, A. Ozguler, F. Ruiz, K. Beaumont, D. Brault, E. Sandt, M. Goldberg, M. Zins

**Affiliations:** ^1^Inserm UMS 011, Population-based Epidemiological Cohorts, Villejuif, France; ^2^Université Paris-Saclay, UVSQ, Univ. Paris-Sud, Inserm, Équipe d'Épidémiologie respiratoire intégrative, CESP, Villejuif, France; ^3^ClinSearch, Malakoff, France; ^4^Luxembourg Institute of Health, Luxembourg, Luxembourg; ^5^Integrated Biobank of Luxembourg (IBBL), Dudelange, Luxembourg; ^6^Faculty of Medicine, University of Paris, Paris, France

**Keywords:** biorepository, blood samples, urine samples, DNA, biobanking methodology, population-based cohort, longitudinal studies, observational studies

## Abstract

“General-purpose cohorts” in epidemiology and public health are designed to cover a broad scope of determinants and outcomes, in order to answer several research questions, including those not defined at study inception. In this context, the general objective of the CONSTANCES project is to set up a large population-based cohort that will contribute to the development of epidemiological research by hosting ancillary projects on a wide range of scientific domains, and to provide public health information. CONSTANCES was designed as a randomly selected sample of French adults aged 18–69 years at study inception; 202,045 subjects were included over an 8-year period. At inclusion, the selected participants are invited to attend one of the 24 participating Health Prevention Centers (HPCs) for a comprehensive health examination. The follow-up includes a yearly self-administered questionnaire, and a periodic visit to an HPC. Procedures have been developed to use the national healthcare databases to allow identification and validation of diseases over the follow-up. The biological collection (serum, lithium heparinized plasma, EDTA plasma, urine and buffy coat) began gradually in June 2018. At the end of the inclusions, specimens from 83,000 donors will have been collected. Specimens are collected according to a standardized protocol, identical in all recruitment centers. All operations relating to bio-banking have been entrusted by Inserm to the Integrated Biobank of Luxembourg (IBBL). A quality management system has been put in place. Particular attention has been paid to the traceability of all operations. The nature of the biological samples stored has been deliberately limited due to the economic and organizational constraints of the inclusion centers. Some research works may require specific collection conditions, and can be developed on request for a limited number of subjects and in specially trained centers. The biological specimens that are collected will allow for a large spectrum of biomarkers studies and genetic and epigenetic markers through candidate or agnostic approaches. By linking the extensive data on personal, lifestyle, environmental, occupational and social factors with the biomarker data, the CONSTANCES cohort offers the opportunity to study the interplays between these factors using an integrative approach and state-of-the-art methods.

## Introduction

Large-scale, prospective observational cohorts have become essential resources for investigation into the causes of many diseases, especially common multifactorial diseases with multiple environmental and genetic determinants (1–4). When they are based on samples representative of the general population, prospective cohorts may also be used for descriptive and epidemiological surveillance purposes. “General-purpose cohorts” in epidemiology and public health are designed to cover a broad scope of determinants and outcomes, in order to answer several research questions, including those not defined at study inception. As just few of many examples, cohorts such as UK Biobank (5), the JANUS biobank (6), the German National Cohort (7) constitute research platforms open to the research community for developing various projects. They offer the opportunity to test several and various scientific hypotheses, and to study the complex interactions of environment, behaviors, and genetics on diseases. They bring new knowledges useful in improving evaluation of personal risk, identifying mechanisms of disease, and directing potential targets for behavior and medical interventions, prevention and treatment. In this context, the general objective of the CONSTANCES project is to set up a large population-based cohort that will contribute to the development of epidemiologic research by hosting ancillary projects on a wide range of scientific domains, and to provide public health information (8, 9). The present paper briefly describes the design of the CONSTANCES Cohort, extensively describes the processes related to the biobank i.e., from collection to distribution, gives an overview of the on-going research projects and discusses the future research opportunities.

## Design and Description of the Constances Cohort

CONSTANCES is a population-based general-purpose cohort designed as a randomly selected sample of the French adults aged 18–69 years at study inception. The source population of CONSTANCES was restricted to salaried workers, professionally active or retired and their family (more than 85% of the French population, i.e., ~56 million people). Overall, in February 2020, 202,045 subjects have been included. The sampling was done within the database of the National Pension Insurance Fund (CNAV: Caisse Nationale d'Assurance Vieillesse). The participation rate at the enrolment in the cohort was 7.3%. During the follow-up, the return rate of annual questionnaire varied slightly depending on the year and was of 75% on average. Regarding loss to follow-up, almost none of the people included in CONSTANCES will be permanently lost to follow-up, since the participants will be followed passively through the national administrative databases. The real lost to follow-up are participants who decided to quit; as of February 2020, there were 194 withdrawals of consent (10).

**Figure 1:**
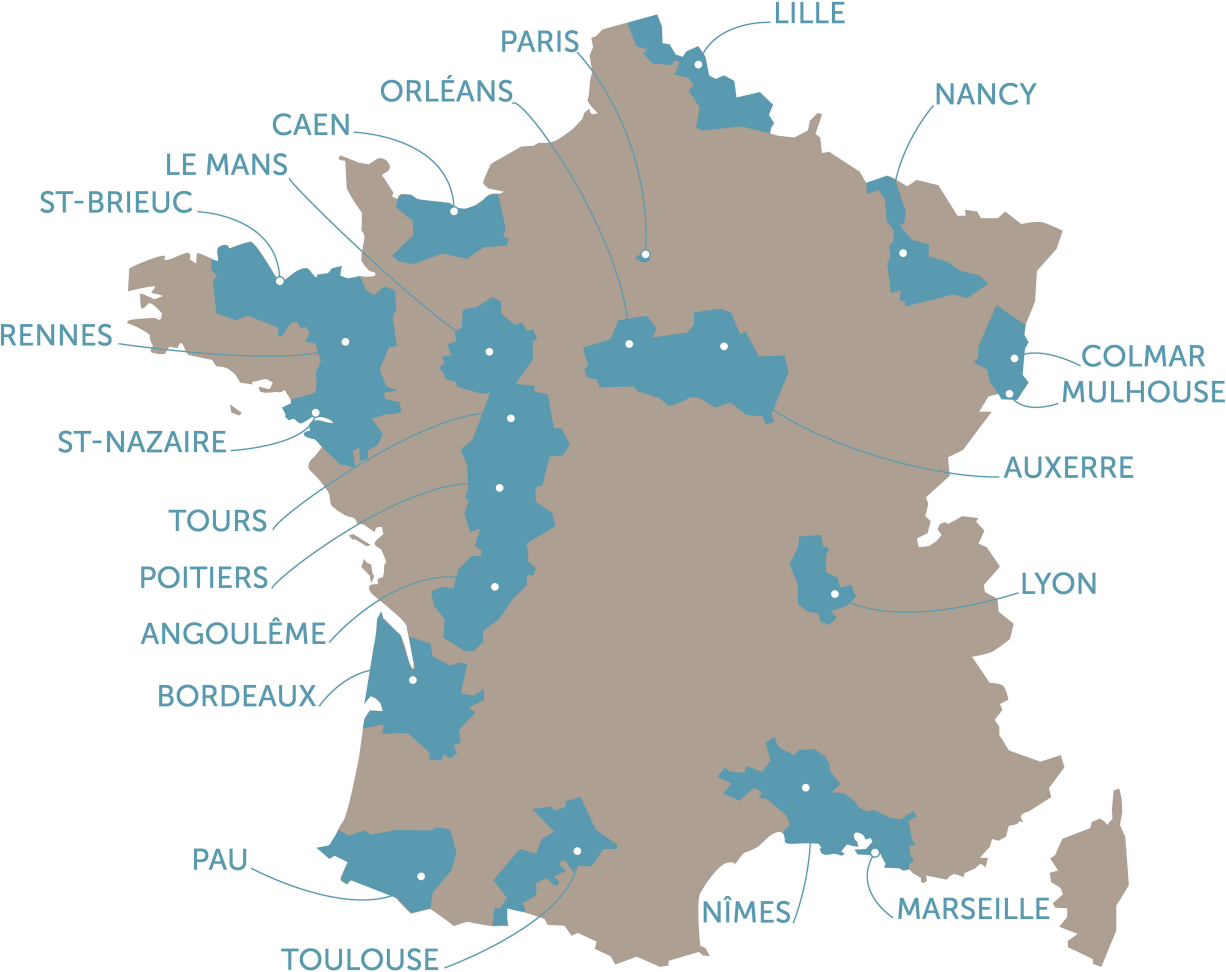
Location of the 24 participating Health Prevention Centers (HPC, Centre d'examens de santé) throughout the 21 cities metropolitan France.

At inclusion, the selected participants are asked to complete questionnaires (11) and are invited to attend one of the 24 participating Health Prevention Centers (HPCs, Centre d'examens de santé) located in 21 cities throughout metropolitan France (Figure 1) for a comprehensive health examination including biometry (weight, height, waist and hip circumference), blood pressure, electrocardiogram, vision, audition, and spirometry. Blood and urine samples are collected for measuring biological parameters related to liver or renal functions, dyslipidemia, glucose metabolism, whole blood cell counts and to communicable diseases such as Hepatitis B and C, HIV, sexual transmitted diseases. For participants aged 45 years and older, a specific work-up of functional, physical, and cognitive capacities is also performed.

An active follow-up includes a yearly postal or web-based self-administered questionnaire, and a complete 4-year follow-up to an HPC including an health examination. Moreover, data are regularly extracted from the French administrative and health national databases, including hospital discharge summaries, visits to health professionals, medication and other prescriptions, severe chronic diseases, sick leaves, handicaps, disabilities and injuries, cause of death, as well as social and demographic characteristics, socioeconomic and employment status. Extensive procedures have been developed to use the national healthcare databases to allow identification and validation of diseases over the follow-up (Table 1).

**Table 1:** Crude prevalence of major health outcomes during the 2012–2017 period in 132,628 participants.

	At least one current long-term disease (ALD^*^) in all participants (***n*** **=** **132,628)**							
	**Between 2012 and 2017**		**In 2015**		**In 2016**		**In 2017**	
**ALD categories**	***n***	**‰**	***n***	**‰**	***n***	**‰**	***n***	**‰**
1- Disabling stroke	706	5.3	488	3.7	546	4.1	613	4.6
5- Severe heart failure, arrhythmias, valvular cardiomyopathy, congenital cardiomyopathy	1,257	9.5	854	6.4	1,005	7.6	1,182	8.9
6- Active chronic diseases of the liver and cirrhosis	487	3.7	368	2.8	367	2.8	382	2.9
7- Primary severe immunodeficiency requiring long-term treatment, infection by HIV virus	356	2.7	332	2.5	346	2.6	348	2.6
8- Diabetes mellitus types 1 & 2	3,155	23.8	2,478	18.7	2,780	21.0	3,098	23.4
13- Coronary heart disease (CHD)	2,244	16.9	1,700	12.8	1,939	14.6	2,180	16.4
16- Parkinson's disease	182	1.4	131	1.0	150	1.1	179	1.3
19- Chronic nephropathy and primary nephrotic syndrome	221	1.7	171	1.3	186	1.4	204	1.5
22- Severe evolutive rheumatoid polyarthritis	507	3.8	391	2.9	434	3.3	466	3.5
23- Long-term psychiatric conditions	2,659	20.0	2,095	15.8	2,266	17.1	2,336	17.6
24- Ulcerative colitis and evolutive Crohn's disease	581	4.4	501	3.8	507	3.8	523	3.9
25- Multiple sclerosis (MS)	264	2.0	231	1.7	245	1.8	259	2.0
27- Severe ankylosing spondylitis	635	4.8	516	3.9	558	4.2	603	4.5
30- Malignant tumors, malignant lymphatic or haematopoietic tissue	6,590	49.7	4,577	34.5	4,841	36.5	5,231	39.4

* *ALD (“Affection Longue Durée”): serious chronic disease*.

Numerous procedures and controls have been set up for the CONSTANCES study in order to ensure that the data is of high quality and reproducible and to allow appropriate assessment of potential measurement biases. For more details see Ruiz et al. (12).

### Access to the Resource

French or foreign research teams wishing to make use of the CONSTANCES cohort infrastructure must submit an application (13). Projects may only use the available data or biological materials and/or collect additional data for a specific purpose. Cohort data access applications are submitted in the context of a permanent Call for Proposals. Access to the Infrastructure is governed by the CONSTANCES Charter, which specifies the rights and responsibilities of the research teams whose projects have been accepted.

Applicants are invited to draft a scientific protocol of their research project specifying the scientific objectives, the method, the expected results, the data requested (and the justification for their use), and, if applicable, the elements related to additional data collection. The projects are then examined by the CONSTANCES International Scientific Committee and, where applicable, by its Ethics Review Board; authorizations are issued by the Institutional Steering Committee comprised of CONSTANCES's partner organizations. Applications must be submitted to the French legal authority for personal data processing, before the required data are sent to the researcher in charge of the project.

To ensure that data confidentiality and security are maintained, the CONSTANCES team generates a specific study number for each cohort participant included in a research project. Moreover, a log of variable requests submitted for a given project is kept to ensure that no-one has access, even after submitting multiple variable requests, to the entire CONSTANCES database.

### The CONSTANCES Biobank

The collection of biological samples for the CONSTANCES Biobank began in June 2018. A 3-month pilot phase preceded the gradual scale-up and extension of collection to all participating collection sites. As of February 29, 2020 29,589 participants were included. Table 2 represents the main sociodemographic characteristics of the whole sample of the CONSTANCES Cohort and of those of the Biobank participants in February 2020. Biosampling of the 83,000 donors is scheduled to be completed by June 2022 at the earliest (due to the interruption of collections during the COVID 19 lockdown). The constitution of the biobank is still in progress: it is proposed to each participant of the cohort when they come to the examination center. The acceptance rate is 94% so far, but we have not yet studied the characteristics of those subjects who refuse the biobank.

**Table 2:** Main sociodemographic characteristics of the whole CONSTANCES sample and of the biobank participants^*^.

	**Whole sample (*****n*** **=** **202,045)**		**Biobank (*****n*** **=** **29,589)**	
	**Per cent**	***n***	**Per cent**	***n***
**Age, year**				
18–29	12.9	25,984	9.20	2,716
30–39	20.5	41,423	20.3	6,019
40–49	23.2	46,845	23.8	7,045
50–59	21.3	43,036	22.7	6,716
60 and more	22.1	44,757	24.0	7,093
**Gender**				
Men	46.4	93,730	48.3	14,298
Women	53.6	108,315	51.7	15,291
**Diploma**				
No diploma or lower	23.9	48,271	18.8	5,555
than high school	16.0	32,408	14.1	4,175
High school	25.3	50,992	25.8	7,646
College university	31.9	64,399	37.9	11,229
Other diploma	0.2	523	0.2	69
Missing data	2.70	5,452	3.1	915
**Marital status**				
Single (never married)	27.0	54,663	24.1	7,136
Married, civil partnership	57.6	116,353	60.7	17,967
Divorced, separated	10.3	20,903	9.9	2,919
Widower	1.9	3,758	1.9	557
Missing data	3.1	6,368	3.4	1,010
**Geographical origin**				
France	87.3	176,348	88.4	26,161
DOM-TOM	0.9	1,853	0.8	229
European union	4.0	8,126	3.9	1,167
North Africa	2.7	5,326	2.0	588
Sub Saharan Africa	1.1	2,208	0.7	214
Asia	0.8	1,638	0.7	196
other part of the world	1.0	2,048	0.9	277
Cannot answer	0.1	195	0.1	28
Missing	2.1	4,303	2.5	729

* *Data computerized and cleaned in February 2020*.

**Figure 2:**
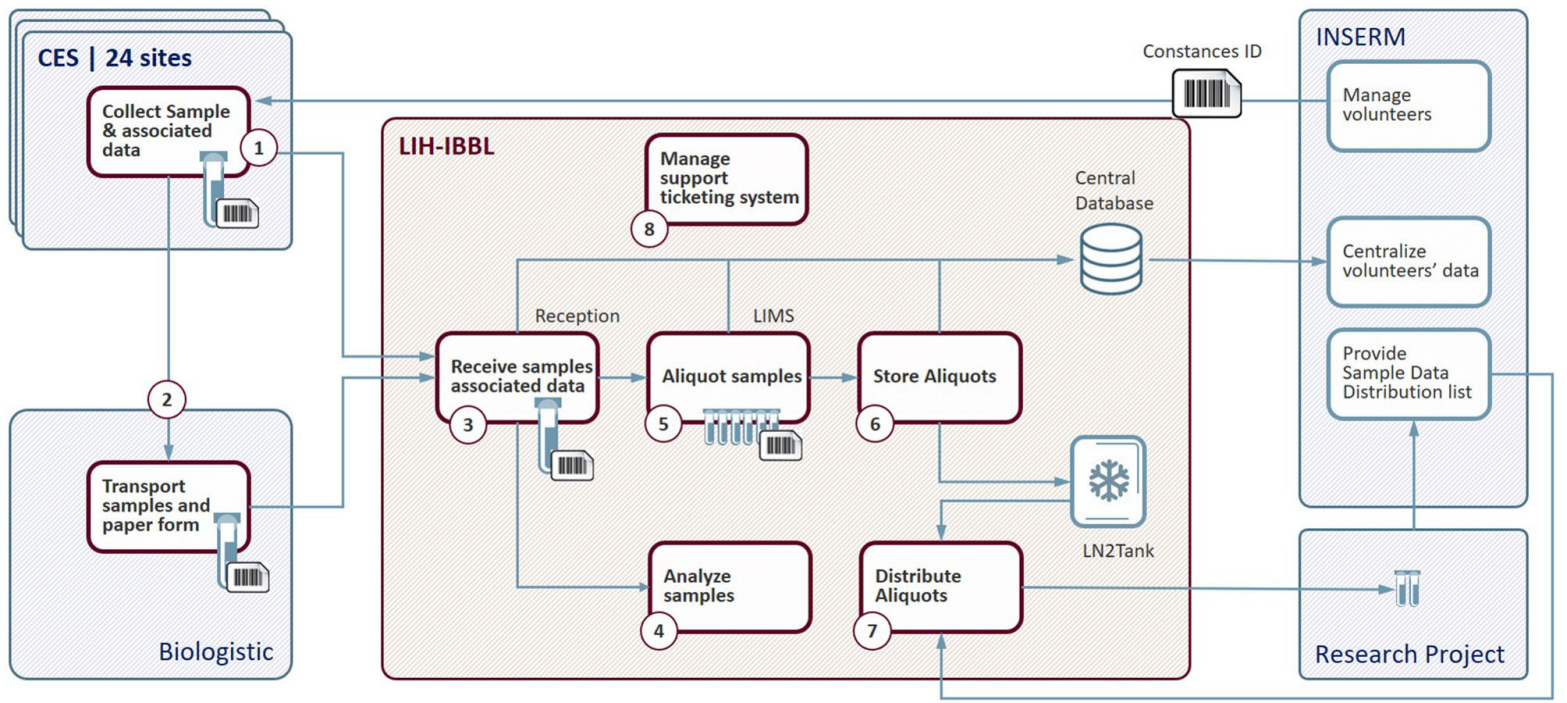
Biobank flow chart. CES, Centre d'Examens de Santé (Health Prevention Center); LIH-IBBL, Luxembourg Institute of Health- Integrated BioBank of Luxembourg; LIMS, Laboratory Information Management System; LN2, liquid Nitrogen; INSERM, National Institute of Health and Medical Research.

The management of the Cohort CONSTANCES Biobank has been entrusted to IBBL (Integrated Biobank of Luxembourg) (14). Within this framework, IBBL ensures the coordination of all the technical operations constituting the CONSTANCES Biobank (Figure 2).

The CONSTANCES team has the overall responsibility for the project and supervises the operations. A steering committee (Comité de pilotage, COPIL) made up of IBBL and INSERM representatives meets regularly to take stock of the situation, ensures project follow-up and makes all required decisions. Technical meetings, bringing together specialists from each entity, aim to discuss the day-to-day operations, identify the weaknesses and propose actions to improve or solve practical problems encountered.

#### Biosampling

Collections of biological samples are carried out in the 24 Health Prevention Centrers) spread all over France, from Monday to Friday.

The blood and urine collections, intended for the CONSTANCES Biobank, are performed immediately after the collection of tubes intended for the Health Preventive Examination (HPE).

Three blood tubes are collected:

- Without additive, Serum separator clot activator tube,
- Lithium Heparin, separator tube,
- K2EDTA tube.

To avoid any interference due to anticoagulants, a purge tube is collected between the tubes devoted to the standard health examination and those intended for the CONSTANCES biobank.

The urine is collected in a sterile urine cup and then transferred with an integrated transfer device in two additive-free transport tubes.

The collection protocol for blood samples is as follows:

- Signature of informed consent,
- Fasting volunteer for 12 h,
- Sampling between 7:00 and 10:00 A.M.,
- Centrifugation of serum and lithium heparin plasma tubes within 30 and 45 min maximum after sampling,
- Storage of the tubes at +2/+8°C until pickup by the carrier.

The collection protocol followed for urine samples is the same as above, except the centrifugation step.

The data related to blood and urine collections is captured in the GeT application. GeT (Greiner eHealth Technologies) is a Greiner BioOne (GBO France, Les Ullis, F 91 941 Courtaboeuf CEDEX) application developed for registration of collection information, adapted to the specific needs of the CONSTANCES Biobank to include collection, primary processing, temporary storage and shipment information. Data from GeT are transferred to IBBL reception application on a daily basis.

#### Shipment

The shipment of the biological samples to IBBL is ensured by a subcontractor specialized in health product delivery (Biologistic, a subsidiary of Chronopost, F 75 000 Paris).

Samples are packed and transported according to ADR (European Agreement concerning the International Carriage of Dangerous Goods by Road) and, additionally, to requirements specific to the Constances biobank.

BioLogistic:

- Supplies the 24 HPCs with reusable triple packaging to ensure proper sample packing,
- Delivers samples to IBBL, from Tuesday to Saturday, within 30 h following the collection, D+1 between 9:00 and 10:00 A.M.,
- Ensures transport at a controlled and monitored temperature of +2–8°C, including during transit phases,
- Guarantees a replacement vehicle in the event of a serious problem.

#### Processing

When arriving at IBBL, samples are received, verified and registered in the Reception application.

Operators:

- visually inspect the tube integrity (readable barcode, undamaged tube, sufficient blood or urine volume),
- Register the tube by scanning it in the Reception application (receipt time stamp),
- Control if the number of tubes received corresponds to the number of tubes expected (information coming from GeT),
- Verify conformity of the transport conditions (packing, temperature),
- Sort the tubes per sample type for sample processing.

IBBL implemented automated solutions to perform the aliquoting of the different samples as detailed in the sample scheme (Table 3). Automate redundancy and manual back up procedures were developed in parallel to ensure processing continuity in case of major technical issue.

**Table 3:** Aliquoting scheme.

**Sample type**	**Primary container**	**Derivative**	**Aliquoting Scheme**	**Container type**	**Long term Storage**
Blood	CAT tube	Serum	6 × 0.4 mL	FluidX 0.7 mL	LN2 vapor
	HEP tube	Plasma heparinized	6 × 0.4 mL	FluidX 0.7 mL	LN2 vapor
	EDTA tube	Plasma EDTA	6 × 0.4 mL	FluidX 0.7 mL	LN2 vapor
		Buffy-coat	2 × 0.45 mL	FluidX 0.7 mL	LN2 vapor
Urine	Additive-free tube	Urine	6 × 1.9 mL	FluidX 2.0 mL	LN2 vapor

These automated solutions guarantee a 36-h cryopreservation timeframe following sample collection and cope with the average load of 100–120 volunteers per day. The operations take place at room temperature within a maximum processing time of, respectively, 1 h 30 for urine, 2 h00 for serum and heparin plasma; and 3 h 00 for EDTA plasma and buffy coat.

Aliquoting of serum, heparinized plasma and urine tubes is performed on two Beckman Coulter AutoMate 1250 platforms (Beckman Coulter France, F 93420 Villepinte). The EDTA tubes are centrifuged upon receipt and plasma and buffy coat are aliquoted via a TECAN Freedom Evo 200 platform (TECAN Group Ltd, CH 8708 Männedorf). Both platforms operate with SBS format racks and 2D barcoded tubes.

For each parent tube aliquoting, from each platform, an output file with all relevant information per child aliquot is produced and imported in the IBBL Laboratory Information Management System (LIMS): Operator, parent ID number, bottom ID barcode, rack ID number, position in the rack, volume, sample type, time stamp and platform name.

Samples are aliquoted in FluidX tubes which were selected taking into account several criteria:

- Suitable to cryostorage (liquid nitrogen vapor condition),
- External thread,
- High-quality virgin polypropylene,
- Biocompatible (free from RNAse/DNAse, endotoxin, phthalate, biocides, no heavy metals, …),
- Tri-coded tubes (2D barcode on bottom, 1D barcode and human readable on side),
- Storage in cryoboxes allowing storage optimization (low profile, 10 × 10 and 14 × 14 formats).

For each serum and plasma sample Haemolysis, Lipaemia and Icterus index (HLI) measurement is performed by COBAS Integra 400 Plus (Roche Diagnostics SA CH 6343 Rotkreuz).

Data related to biosampling processing, annotations and results is recorded in the LIMS.

#### Storage

Once aliquoted, the cryotubes from the SBS racks (8 × 6 and 12 × 8) are sorted in 2 mirror selections and each cryotubes selection is consolidated in a different cryobox to guarantee the mirror and optimize the storage capacity. This consolidation is performed at 2–8°C prior initial freezing in a temperature monitored CryoCart maintaining optimum liquid nitrogen vapor conditions during sample handling. Samples are frozen within 36 h following the collection. Mirror collection is implemented by dividing the child samples in two different cryoboxes and also storing these two cryoboxes in two different cryogenic freezers.

The new position of each aliquot is automatically recorded and updated in the IBBL Laboratory Information Management System (LIMS) by scanning the consolidated cryobox. The storage location of the cryobox is indeed registered in the LIMS, providing the following pathway: tank ID number, sector ID number, tower ID number, Box position in the tower and Cryobox ID number.

The samples are stored in MVE Heco 1800 (MVE Bio) cryogenic freezers containing liquid nitrogen under the carousels hosting the towers with the cryoboxes in which the cryotubes are stored. The samples are stored in liquid nitrogen vapor at a temperature guaranteed by the manufacturer of −190°C (+/−5°C). Two parallel and independent alarm systems monitor the cryogenic freezers, with personnel on duty 24/7. One backup cryogenic freezer remains empty for emergency transfer and/or planned maintenance. Storage of samples from the planned 83,000 donors represents 12 MVE Heco 1800 cryogenic freezers.

#### Sample Distribution

Request for distribution of biological samples for a research program is submitted to the Principal Investigator (PI) of the CONSTANCES Cohort and evaluated by the international scientific council, according to pre-established criteria (12).

If necessary, adjustments to the project, concerning either the methodology (feasibility) or the scientific objectives, might be required. If the project is accepted, the CONSTANCES team provides IBBL with a list of CONSTANCES ID numbers corresponding to the applicant's selection criteria (on the basis of clinical data, para-clinical data and questionnaires).

Upon receipt, IBBL generates a working list, called picklist, containing the ID numbers of the aliquots and their storage location. The aliquot picking is performed in a CryoCart, in order to maintain a working environment below −150°C.

The samples are picked up, scanned in the LIMS, stored in SBS racks or cryoboxes, and QCed before release for redistribution. Shipment is performed either in Dry-Shippers (Liquid nitrogen vapor) or in dry ice. An electronic manifest listing the samples with their storage location is generated from the LIMS, and a paper manifest is attached to the parcel(s). Sample redistribution details are tracked in the LIMS (samples redistributed, package and package content, consignee, courier, tracking number, shipment date…).

#### IT Infrastructure

The infrastructure of the Business Information System (BIS) consists of three main applications:

- The GeT solution (Greiner Bio One's software package) manages the collection of data in the Health Prevention Centers: collection, pre-analytical processing, temporary storage and shipment to IBBL.
- The Reception application that controls and centralizes all the data coming from the different sources: GeT application, BioLogistic file exchange (transport status, temperature curve status).
- The Laboratory Information Management System solution (LabVantage software package; LabVantage France F 92110 Clichy) that hosts the main database and gathers data from the Reception Interface, as well as data from sample processing, analysis, annotation and storage.

**Figure 3:**
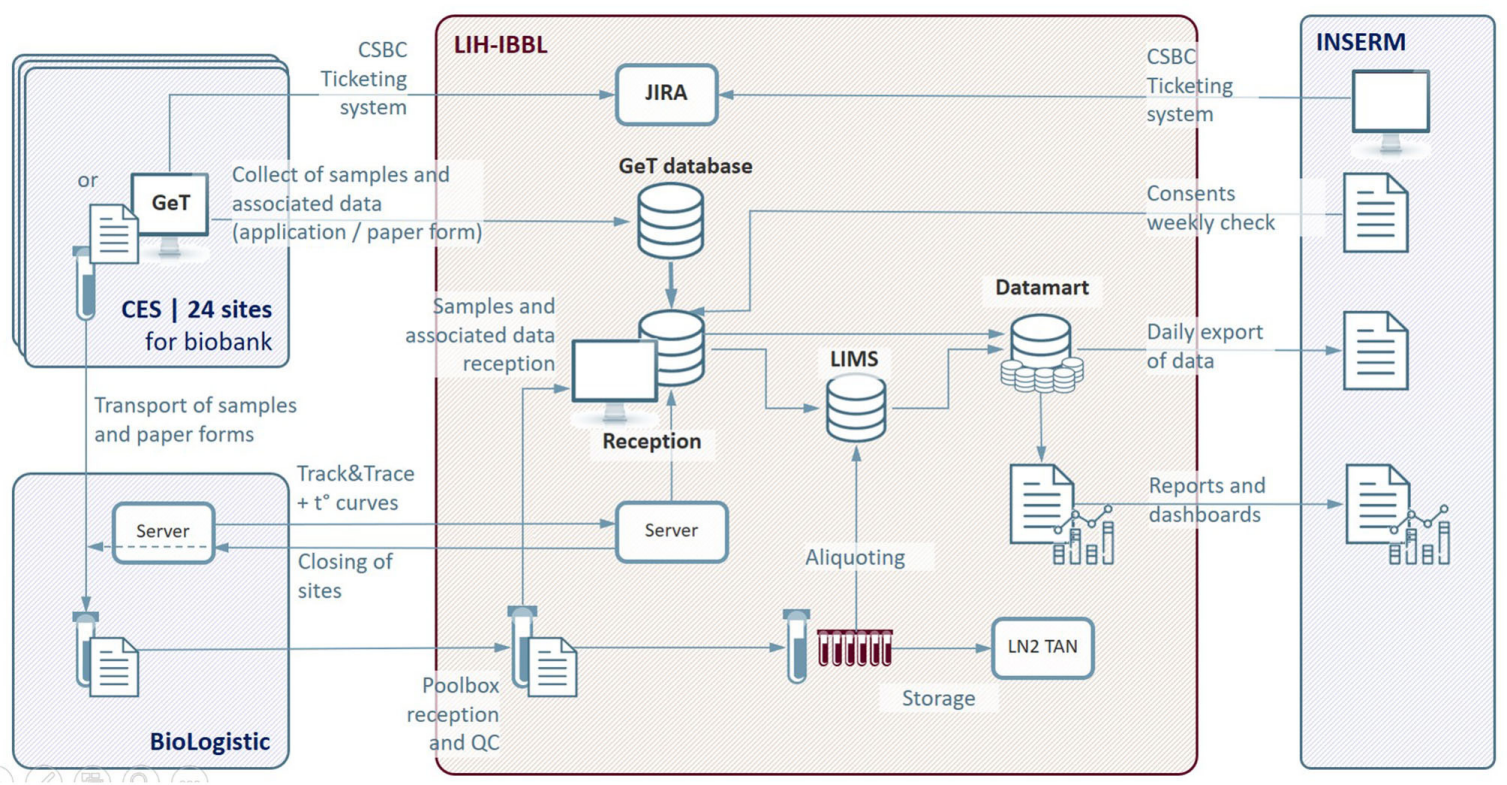
IT architecture of the Constances Biobank Information System. CES, Centre d'Examens de Santé (Health Prevention Center); CSBC, Constances Service Desk; GeT, Greiner eHealth Technologies; LIH-IBBL, Luxembourg Institute of Health- Integrated BioBank of Luxembourg; LIMS, Laboratory Information Management System; LN2, liquid Nitrogen; INSERM, National Institute of Health and Medical Research; JIRA, bugs and incident management system; QC, Quality Control; t°, temperature curves.

The schematic diagram of the information treatment infrastructure is shown in Figure 3.

The main functions of the Information Treatment Infrastructure are summarized below:

- Biosampling data entry at the Health Prevention Center in GeT and transfer to the Reception application,
- Sample reception registration and Quality Control in the Reception application,
- Sample processing data exchanges and capture in LIMS:
  ∘ Management of aliquoting robots interfaces,
  ∘ Recording of identification data during aliquoting operations,
  ∘ Storage location registration of each aliquot and update of inventory after each input/output operation,
  ∘ Inventory status,
  ∘ Samples retrieval traceability,

- Stock management of consumables used by Health Prevention Centers via an home-made calculator,
- Data traceability and exchanges:
  ∘ sample quality annotations, non-conformities and history of each aliquot in LIMS,
  ∘ Data security, encrypted communication,
  ∘ Reporting and dashboarding: edition and consultation of periodic reports on the number of inclusions, the number of stored samples (specimens and aliquots), non-conformities observed during the process,

- Centralized Communication interface for all the different CONSTANCES Biobank actors, CONSTANCES Service Desk (CSBC).

All the data relating to the CONSTANCES Biobank, collected at different levels and by different tools, are consolidated in a single database. These data can be accessed through a data warehouse allowing the grouping and structuring of data from different sources of information; this data warehouse makes it possible, in particular, to base all management, statistical and quality control reports.

All of the “technical” data relating to the biobank management process, updated daily, are transferred regularly to CONSTANCES Information Treatment team to be consolidated with the clinical and paraclinical data collected elsewhere. Thus, the CONSTANCES staff has an overview of all the information sets collected.

Access to the various interfaces and servers is limited to authorized persons in each organization (Health Prevention Centers, IBBL and INSERM). The level of authorization is defined according to the responsibilities of each person.

#### Quality Management System (QMS)

IBBL's Quality Management System is conceived to be compliant with the requirements of:

- ISO 9001:2015 Quality management systems – Requirements,
- ISO 17025:2017 General requirements for the competence of testing and calibration laboratories,
- ISO 20387 Biotechnology — Biobanking — General requirements for biobanking.

IBBL currently holds an ISO 9001 certification and an ISO 17025 accreditation.

For the CONSTANCES Biobank, IBBL applies its general certified and accredited QMS and in addition has implemented process specificities.

Each step of the CONSTANCES biobank process is carefully described in dedicated Personalized Standard Operation Procedures as listed in Table 4.

**Table 4:** Constances Biobank Project Specific Operating Procedures.

**Operational project processes**	
	CONSTANCES – Protocole opératoire des CES
	CONSTANCES – Operational activities of IBBL
	CONSTANCES – Project management and reporting
	CONSTANCES – IT infrastructures
	CONSTANCES – Quality plan
	CONSTANCES – Reporting and data exchange
	CONSTANCES – Sample destruction and distribution
	CONSTANCES – Non-conformities management

IBBL is in charge of implementing the CONSTANCES quality plan. The quality objectives are defined in conjunction with the CONSTANCES team, which validates all operating procedures. Objectives and improvement paths are defined during periodic quality review meetings. An inventory of non-conformities is provided regularly, reviewed and analyzed. Correctives and/or preventive actions are defined, implemented and efficiency verified.

#### Quality Assurance

In order to ensure that the specifications are met and that the biological samples are of high quality, periodic checks shall be carried out.

#### Traceability

A significant set of data is collected during the whole process and corresponding variables are summarized in Supplementary Table 1 (Supplementary Material).

#### Non Conformity Control

Communication between IBBL and the Health Prevention Centers is ensured via the CONSTANCES Service Desk, CSBC.

The CSBC is used to communicate and document some non-conformities and/or deviations detected from both sides, such as:

- Transport delay
- Missing data entry

Periodic monthly reports of non-conformities overall and for each inclusion site are published. They are reviewed at each Steering Committee (COPIL) and, if necessary, corrective measures are implemented. Table 5 lists the main non-conformities observed and their frequency.

**Table 5:** Relevant non-conformities and their frequency.

	**Specimens (tubes)**		**Aliquots**	
	***N***	**%**	***N***	**%**
**Total number of items**				
Specimens (tubes)	**163,690**			
Aliquots			**735,610**	
**Main non-conformities**				
Late storage	3,066	1.87	15,448	2.10
Incorrect centrifugation parameters	2,506	1.53	14,231	1.93
Prolonged centrifugation delay	2,323	1.42	12,563	1.71
Low volume - manual processing	1,194	0.73	57	0.01
Potentially not coagulated	922	0.56	5,086	0.69
Insufficient volume	433	0.26	0	0.00
Menstruations	331	0.20	965	0.13
Incorrect temporary storage conditions	289	0.18	1,553	0.21
Empty tube	172	0.11	0	0.00
No purge	113	0.07	562	0.08
Tube not centrifuged by HPC	67	0.04	354	0.05
Tube expired	46	0.03	262	0.04
Broken tube	22	0.01	0	0.00

*The number and proportion (%) of observed non-conformities have been reported for the tubes collected and the aliquots generated from each tube*.

#### Process Control

Each step of the process is subject to different quality controls, on a daily basis, as detailed through the Personalized Standard Operating Procedures (collection, transport, processing of biological samples, storage of samples, distribution).

Periodic verification of sample inventory: twice a year

Security systems concern more specifically the storage area:

- Two redundant alarm systems for detection of temperature excursions, liquid nitrogen level over filling and power supply faults affecting the cryogenic freezers,
- An oxygen alarm system related to staff safety,
- Ventilation with a 10x automated forced extraction in case of oxygen alarm activation,
- UPS power supply and emergency power supply by diesel generator.

#### Reporting

IBBL provides the CONSTANCES team with periodic monthly reports:

- Status of inclusions for each Health Prevention Center and for whole:
  ∘ number of volunteers included,
  ∘ details for each type of biological specimens received at the biobank,
  ∘ details for each type of aliquots made for each type of biological specimen,

- Status of non-conformities for each Health Prevention Center,
- Inventory status,
- Status of input/output of biological samples (if any sample distribution within the month),
- Status of sample destruction (if any sample destruction within the month).

**Figure 4:**
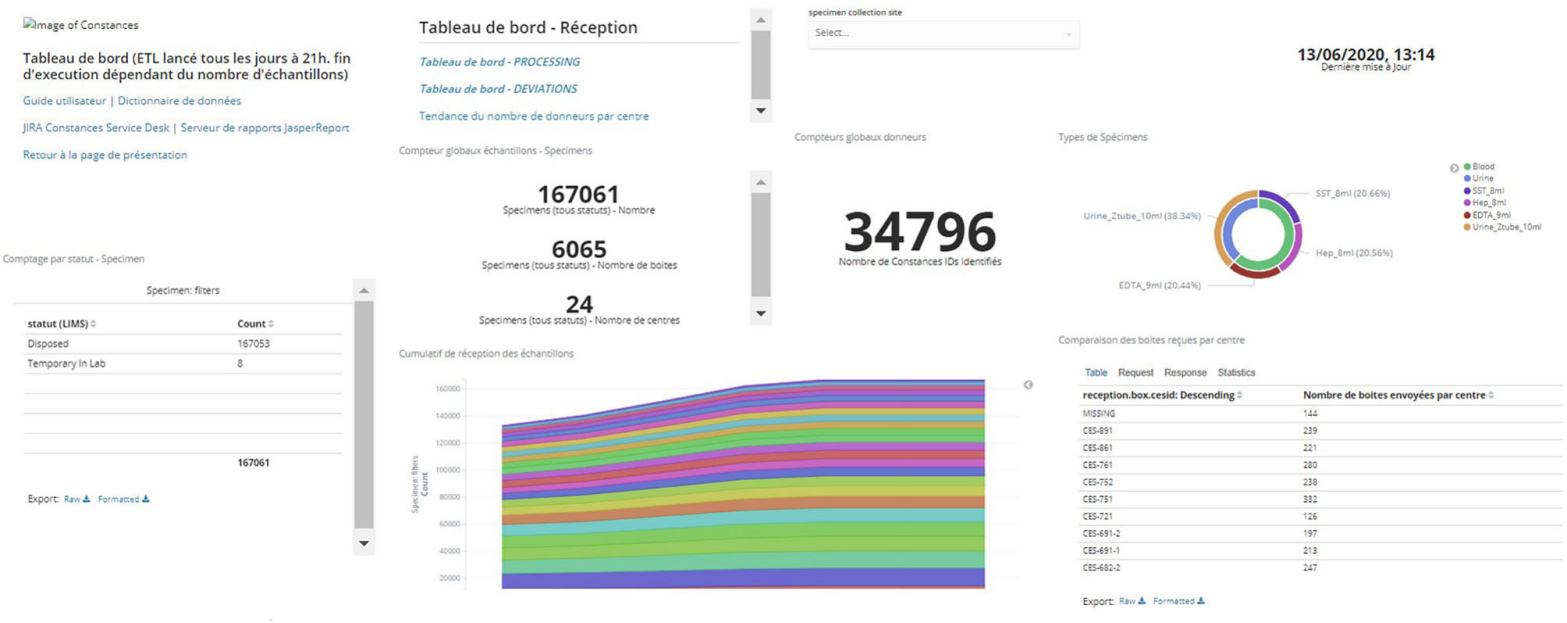
Example of data warehouse dashboard: Reception dashboard.

These reports are available in a format that allows for subsequent reprocessing as well as a direct visualization mode for the Steering Committee. They can be generated and graphically displayed in real time through the data warehouse interface that can be consulted by authorized persons on the IBBL portal as shown in Figure 4. This figure is an example among others of the multiple dashboards used to follow the biobanking process; it represents the evolution of the number of samples collected for each inclusion site as a function of time and the proportion of different types of specimens (serum, plasma, buffy coat, urine). Other dashboards are available, e.g., to monitor the proportion of non-conformities observed.

#### Monitoring and Communication With Inclusion Sites

Two members of the CONSTANCES team are dedicated to training, information and communication with the medical and para-medical staff of each Health Prevention Center. They intervene during the implementation of the cohort and answer daily practical questions from each Health Prevention Center in close collaboration with the IBBL staff.

A CONSTANCES Service Desk (CSBC) has been specifically developed by IBBL in the form of a web portal. The CSBC consists of one interface accessible to the service desk customers and IBBL to enter requests and get information on the progress of requests. It is used to track requests from the Health Prevention Center, IBBL and the CONSTANCES team (e.g., transport, collection, data processing and other problems) and solve support requests (also called tickets).

Epidemiological research assistants (ARE: attaché (e) de recherche épidémiologique, Clinsearch, F 92240 Malakoff) periodically visit the Health PreventionCenter to ensure that the procedures in place are being followed. They ensure the traceability of the equipment used by the Health Prevention Center. They can pass on information from IBBL on the non-conformities observed. They collect, as necessary, information on the progress of critical operations (e.g., on the status of stocks of consumables for blood and urine samples).

#### Management of Informed Consents

Informed consent specific to the CONSTANCES biobank is collected upon arrival at the Health Prevention Center. These consents are then validated by the CONSTANCES staff, which ensures that all required information is available and recorded. Until validation of the consents, related biological samples are quarantined and cannot be distributed to researchers.

Weekly, the CONSTANCES staff sends IBBL a file of CONSTANCES ID numbers for which consents have been validated to ensure the volunteers have given or withdrawn their consent. This information is recorded in IBBL's Reception database. Then, concerned samples can be used for research purposes or may be destroyed (in case of a consent withdrawal).

## Discussion

### Biobank

The CONSTANCES Biobank is a project associated with the CONSTANCES Cohort, dedicated to the study of large populations and 202 045 participants were included in the cohort from 2012 to 29 February 2020. For many reasons, the biobank was set up late, starting in June 2018 for the pilot phase, with the ramp-up taking place at the end of 2018. As a result, blood and urine samples from 83,000 participants will be collected. According to the designers of the CONSTANCES project, the biobank, like the cohort, is an open tool at the service of research, with no preconceived related scientific projects.

#### Representativeness

The representativeness of the population included in the biobank will be verified at the end of inclusion. Until now, the main socio-demographic characteristics of the cohort sample (i.e., the 202,045 already included volunteers) and those of the biobank participants are very similar (Table 2). Only minor differences can be observed: e.g., 18–29 age class: 12.9 vs. 9.2%, 60 and more age class: 22.1 vs. 24.7%, no diploma: 23.9 vs. 18.9%, respectively, no difference is observed according to geographical origin.

The biobank inclusions started only 1 year before the end of the first cohort inclusions: they will continue until they reach the expected 83,000. As a result, the majority of volunteers will be included in the second wave of invitations, 4 years after the first inclusions. Only about a quarter of the volunteers will be able to match the biobank samples with the clinical, para-clinical and environmental data from the first wave of inclusion. This may be a limitation for longitudinal studies. It will also be necessary to check attrition in the second wave, a well-known phenomenon in longitudinal studies over a very long period, and to assess its impact on the representativeness of the biobank.

The relatively small number of biobanked participants compared to other population-based cohorts (UK Biobank, German national cohort.) will limit longitudinal studies based on the occurrence of outcomes to the most frequent diseases, including chronic diseases. Other diseases with low prevalence, such as certain cancers, will not be eligible for research projects because of their small number. This is a limitation frequently encountered and encourages the pooling of resources among several cohorts and biobanks.

#### Design of the Biobank

The design of the biobank was conceived to meet the project of a generalist cohort. Originally, it was envisaged to collect a wide variety of biological materials in addition to those collected in all biobanks: serum, heparinized plasma, EDTA plasma, buffy-coat (Desoxy-nucleic acid: DNA) in order to meet emerging demands for studies on the proteome, metabolome, transcriptome, microbiota (feces), environment (hair, nails). Some of them impose specific collection conditions: dedicated tubes, rapid pre-analytical treatment (transcriptome) or are poorly defined or not subject to consensus (proteome). It was decided to limit the collection to commonly used biological materials that already have a wide range of potential uses, with the possibility of extending this to some of the above-mentioned biological materials for specific programmes. Anticipating future needs for biological material, such as type of sampling, number of aliquots, sample volume, pre-storage conditions, is much more difficult to manage in an open cohort than in a context where research projects are initially clearly defined. These uncertainties have imposed a compromise solution that is inexpensive but offers fewer prospects for development.

Particular attention was paid to the conditions for the collection of biological specimens and the pre-conservation phase (the pre-analytical phase). As it is well-known that many biomarkers degrade more or less rapidly after collection (among them cell lysis, transmembrane exchange, modification of protein conformation), it was necessary to choose between the terms of the alternative:

- Separate serum and heparinized plasma rapidly after blood sampling to avoid the above-mentioned artifacts by centrifugation and immediate storage at +4°-8°C, including during transport, until aliquoting at the biobank. These operations have to be carried out in <36 h.
- Separate the serum from the heparinized and EDTA plasmas immediately, then aliquot on each inclusion site with mini-robots, freeze immediately at −80°C, and then periodically transfer to the biobank the samples frozen in the dry ice.

The first solution was chosen to give priority to the control of the always delicate aliquoting operations, by carrying them out on a single site with well-trained staff, with a limited number of liquid handling robots reducing production and maintenance costs (15). The counterpart of this choice leads to do without the rapid freezing at very low temperatures that can protect certain labile molecules. According to the literature, it has been shown that the number of biomarkers unstable at +4/8°C for <36 h, i.e., the maximum time before freezing at the biobank, was very limited and that a large number of potential demands could thus be met (16).

#### Aliquoting

The number and working volume of the aliquots is also the result of a compromise: the context of biological sample collection is very constrained, since it is integrated into the collection process of the health examination. For ethical reasons, acceptance by the volunteers and their comfort, it was decided to limit the number of blood tubes collected. As a result, a more limited number of aliquots than usual in epidemiological studies is performed. Unfortunately, the objectives are only partially met: during the 1st months we collected only 80–90% of the planned number of aliquots; this led us to reduce the working volume from 0.5 to 0.4 mL to maintain the number of aliquots considering it preferable to privilege the number of possible studies knowing that modern analytical techniques use very low sampling volumes.

#### Method of Preservation

The choice of the conservation mode covers two realities: the choice of temperature and the choice of the type of refrigeration (mechanical freezers or liquid nitrogen vessels); this choice is controversial. Long-term storage in liquid nitrogen vapor was preferred for several reasons: guarantee of temperature maintenance whatever the circumstances [2 weeks minimum of autonomy in case of liquid nitrogen supply failure, highly unlikely except in the event of a disaster (war, etc.)], no modification of the biological materials stored below the vitrification temperature of the water, optimisation of storage (densification), advantageous economic balance compared to other solutions (liquid nitrogen prices are falling, and electricity prices have risen significantly over the decade). In comparison, based on the situation at the time the biobank was designed, mechanical refrigeration solutions had several limitations: potential risk of compressor failure forcing transfers of biological samples to backup freezers or even loss of samples (external temperature maintenance systems are expensive and unreliable over time, dual-compressor freezers were in their infancy), unfavorable energy balance (need for an expensive air-conditioning system to eliminate heat loss, which is also not really environmentally friendly). Automation of storage was not retained, although very attractive; automation solutions for mechanical freezers were available when the biobank was designed, but were particularly expensive, their maintenance was complex, and most of the time required adapted premises, or even a specially designed building. Automated solutions for liquid nitrogen in vapor phase were in their infancy, were particularly expensive and had not proven their reliability over a sufficiently long period of time.

#### Qualification of Samples

Ensuring the quality of biological samples by maintaining the integrity of biomarkers is the first duty of the biobanker. The most critical step is the so-called preconservation or pretreatment phase (often referred to as pre-analytical) (17–20). It has been proposed in the literature to measure biomarkers known to be labile to ensure that samples meet specifications (21). It was also proposed to qualify the samples collected for future use by measuring biomarkers supposed to be used in potential research projects e.g., on cardiovascular disease, kidney disease, carbohydrate metabolism disorders (22). We did not adopt these recommendations for several reasons: we preferred to follow a strict protocol covering the stages from collection of biological fluids to freezing in liquid nitrogen vapor phase. As these predefined conditions are respected, one need only refer to the data in the literature to know whether or not they are sufficient to measure a given analyte (16); for a new biomarker a prior verification step will be necessary to ensure its stability over a given period of time. The measurement of “control” labile biomarkers that can only be measured on a limited number of randomly selected specimens did not seem to us to provide any additional guarantee compared to a standardized protocol whose application is controlled by the collection of relevant traceability data and regular monitoring. On the other hand, it is useful when the conditions of the pre-analytical phase are not known and are not really traced. We have also not retained qualification for future clinical applications, as the search for new biomarkers again requires prior stability verification to ensure that the collection and storage conditions are adequate. It can be estimated that under identical pre-treatment and storage conditions, the literature stability studies data can or will be transposable to the samples in the CONSTANCES biobank.

#### Long Term Sample Stability

This is the key question that arises when using biological samples; to date, there is no unambiguous answer. The assessment of long-term stability presupposes the availability of control material whose stability is itself guaranteed and that the long-term analytical variability is itself measurable and controllable. As far as we know, this is an unreachable dream. Quality control materials whose long-term stability (>10 years) can be guaranteed do not exist, at least not yet. Analytical methods are evolving very rapidly, especially in research, and cross comparisons are very difficult, even unrealistic over long periods of time, even at 10 years or less. Measuring analytical variability over 5 or 10 years with the same analytical system is not really realistic. We were therefore reduced to empiricism: by keeping the conservation conditions constant, it is reasonable to assume that the measurements made at times 0, 1, 2, N with the same analytical system and in a short period of time for example over 10 years are comparable with each other.

In other words, this means that under identical storage conditions for the samples of the same study, the measurement results are comparable between them. However, comparison of these same results with those of other studies carried out under different conditions will require some due diligence.

#### Quality Assurance

A very careful recording of non-conformities is carried out by IBBL at each stage of the process from blood (and urine) sampling to storage. Table 5 shows that the frequency of non-conformities is low compared to the number of tubes collected and aliquots generated. The most frequent non-conformity is due to the transport time not respected by the carrier (2.1%), leading to a delay in storage. This malfunction common in the first few months has been corrected. Logistics is a critical point for all large-scale epidemiological studies, special attention must be paid to it. Non-conformities concerning centrifugation are due to the operating constraints of the HPCs: recommendations for compliance with good practices have been made. Abnormalities in HLI (Hemolysis, Lipemia, Icterus) indices, often mentioned in the literature (23–25), are very limited (data not shown).

#### Data Collection and Information Processing System

The information collection system made available to the inclusion sites is a derivative of those used in medical laboratories. Its original specifications were not really adapted to the needs of a biobank, so we had to redesign it to make it truly operational. On the other hand, the communication application with the inclusion centers (CSBC) developed specifically for the needs of the CONSTANCES biobank proved to be useful and efficient. Biobanks are very special tools that cannot be satisfied with equipment or applications designed for other purposes.

#### Biobanking Solution

This is one of the challenges facing all epidemiology teams. The processing of an extremely large daily volume of biological samples over a long period of time requires an industrial-type organization, far removed from biobanks housing targeted collections, whose samples volume is generally smaller. The question of internalization or outsourcing arises. Some large European biobanks have chosen to internalize their collection, while others have retained control of it, but outsource its management and maintenance. The CONSTANCES team chose an outsourced solution on the basis of multiple criteria: staff skills, adequate infrastructure (buildings, support services, etc.), equipment, guaranteed durability. The guarantee of sustainability is one of the main criteria to be paid attention to; it is difficult to assess. Organizations managing biobanks are not exempt from the risks associated with any economic activity. Investments are heavy, maintenance is costly and activity can vary greatly from 1 year to the next. Unfortunately, it can only be noted that many biobanks have ceased their activity in recent years.

### Ongoing Research Projects

Although designed as a general-purpose cohort intended to host numerous nested projects with a very broad scope, the first orientation of CONSTANCES was the study of occupational and environmental determinants of health (26). These themes are essential areas of research in public health and epidemiology, involving numerous health problems and diverse populations. The 72 ongoing research projects as March 2020 are listed in Table 6 (27). The collection of biological specimens began gradually in June 2018, and the ongoing projects only include as biological markers the parameters collected for the health examination at inclusion and at follow-up in the 24 participating HPCs. Recently, a retrospective study of anti SARS-COV2 serology included 9,144 cohort participants from November, 4 2019 to March, 15 2020 who came in the HPCs before the generalization of the epidemics in France.

**Table 6:** Distribution of the current research projects of the CONSTANCES Cohort (March 2020).

**Research themes**	**%**
Occupational and environmental determinants of health	26.4
Chronic diseases	40.2
Health care, prevention, screening and treatments	15.3
Observation and surveillance	13.9
Health economics	4.2

### Future Research Opportunities

By its thorough system for the follow-up and collection of very diverse information through a variety of methods and data sources on a large representative sample of the adult population, CONSTANCES aims to contribute to a better knowledge of the health of the French population (8). By linking the extensive data on personal, lifestyle, environmental, occupational and social factors with the biobank data, the CONSTANCES cohort offers the opportunity to study the interplays between these factors. This extensive characterization of participants along with the biobank will also open countess opportunities of collaborations in France and abroad.

The advantages of collaboration between large cohorts are unquestionable in genetics regarding e.g., the study of rare diseases or rare alleles, of causality using Mendelian randomization, or of the population selection pressures. However, the paucity of evidence for the clinical utility of genomic testing, and therefore the impossibility to drive transformational change in healthcare, remains a principal barrier to implementation. Indeed, the risk assessment for coronary heart disease, one of the most studied chronic diseases worldwide, do not appear to be improved by the polygenic scores (28). It is noteworthy that the majority of the participants of the genetic studies are of European descent while the African pan-genome contains ~10% more DNA than the current human reference genome (29) leading to a poor generalizability of the polygenic scores across populations (30). Extensive characterization of participants from large sample size cohorts is not enough if representativeness is lacking. Despite a wide variety of phenotypic information and biological samples collected for 500,000 participants and genotypes for 488,377 of them, UK Biobank is not representative of the UK population (5). This lack of representativeness implies to reconsider the idea that associations found are generalizable to all possible target populations, or relevant to public health and clinical medicine (31). Similarly, associations found in CONSTANCES are for example not generalizable to children or to all ethnic groups. However, as CONSTANCES is representative of the French adult population from 18 to 69 years old at inclusion, it offers among others the opportunity to study the genetic and biology of aging or of complex diseases in elderly, a group for whom extensive phenotypic, biological and genetic characterizations are scarce, especially among healthy (32). In order to accelerate and sustain the integration of genomics into healthcare, the collaboration between genetics projects – and therefore between biobanks – across countries is needed (33, 34). As an interesting example, FinnGen is a research project that brings together almost all Finnish biobanks (35). FinnGen plans to utilize 500,000 unique samples collected from a nationwide network of Finnish biobanks more efficiently for research purposes, aiding in the development of better treatments and medications. Among the perspectives opened by “multi-biobank” studies, it should be noted that CONSTANCES participates in several international consortia of population-based cohorts, which will facilitate research on large groups of diverse populations.

Genetics data are only one of the several pieces of a bigger “biological” puzzle which includes transcriptomics, epigenetics, proteomics, and metabolomics. The proteome, transcriptome and metabolome analyses open up opportunities for the identification of new biomarkers for the diagnosis and prediction of clinical events that could be better predictors than traditional ones. Due to the choices we have made, CONSTANCES cannot play yet with all the puzzle pieces. The collected biological samples will allow for a large spectrum of biomarkers studies including hormones, nutrients, key proteins, genetic and epigenetic markers and their interrelations through candidate or agnostic approaches. On the contrary, transcriptomics and metabolomics studies or researches on the microbiome are not possible at this time, but specific programs on this topics or an extension of the biobank are not excluded. Selected examples of studies that could be done yet or in the future with CONSTANCES are as follows. By pooling seven cohorts of multi-ethnic men and women of middle-age, Bancks et al. (36) estimated the long-term absolute risk for developing cardiovascular disease according to multiple glucose categories and by change in category over time. This study highlights the interest of monitoring glucose levels during middle age to prevent incident diabetes by midlife. In a discovery study based on a subsample of women from the 10 year followup of the “Sleep and Health in Women” study, Ljunggren et al. found that obstructive severe apnoea during REM sleep was associated with changed levels of inflammatory and cardiac proteins (37). By using metabolomics profiling of 592 fasting serum metabolites, Menni et al. identified molecular markers and pathways associated with serum electrolyte levels in 1,523 participants from two independent population-based cohorts (38). Felhmann et al. investigated the use of blood-borne miRNAs as potential markers for detecting lung cancer in a cohort of more than 3,000 symptomatic patients and control from case-control and cohort studies. They identified two miRNA signatures of high accuracy, sensitivity and specificity (>80%) to distinguish patients with lung cancer or with early stage (39). Last but not least, epigenome is involved in many different diseases, and play a central role in disease outcome and progression (40). However, epigenome is still far from being fully understood and the clinical practice of epigenetics is still limited. Recently, Langdon et al. reported that thirteen peripheral blood DNAm-based scores were able to predict complex traits with a relatively high proportion of variance explained for smoking, alcohol consumption and BMI in 364 individuals with oropharyngeal cancer from the “Head and Neck 5000” cohort (41). Overall, these results highlight the potential of cohorts coupled with biobank to significantly expand our understanding and application of omics tools in complex traits.

The number of studies integrating multiple omics level is growing, and their results show that including more than one omics level can contribute to better understand the interactions between the omics levels preceding the studied one, and between omics and the environment (42). In the same line, coupling omics with biological markers is of great interest. Thus, after having identified a network of 11 correlated cytokines known to participate in a broad array of immune responses, a genome wide association study (GWAS) of this cytokine network revealed variants with immune, hematological, and cardiometabolic pleiotropy among 9,263 participants from three population-based cohorts (43). The extensive phenotypic and environmental characterization of participants from CONSTANCES along with the biobank samples will allow applying integrative approaches able to study the association between omics and complex traits taking into account exposure to environmental, social and lifestyle factors, and the interplay between these factors. Other key strengths of CONSTANCES are the availability of urine samples and the follow-ups every 4 years with a complete examination. Urine samples will allow performing GWAS of the urinary concentrations of metabolites as done recently in the paper by Schlosser et al. among 1,627 patients with reduced kidney function (44). In order to answer new research questions, CONSTANCES is an adapted framework to collect other fluids such as saliva or feces which will enrich the biobank, or to obtain new data though devices that can be send to the participants' home and send back by them such as electrostatic dust collectors to assess the fungal contamination in dwellings, a preventable risk factor for several respiratory diseases. It should be also noted that among the 200,000 participants 70,000 of them respond to the annual questionnaire online, allowing new questions to be rapidly implemented in response to emergency scientific programs such as those related to the new SARS-COV2 (45).Regarding perspectives in a longitudinal setting, as March 2020, ~18,000 participants had been followed-up at 4-year offering the possibility to develop many new research programs including those related to aging, a process of physiological and molecular changes related to susceptibility to diseases and death. Recently, longitudinal and multi-omics profiling in 106 prediabetic and health individuals revealed personal aging markers and different types of aging patterns or “ageotypes” (46). The identification of endotypes, i.e., subtypes of diseases by integrating clinical, functional and biological characteristics using clustering methods is a rapidly expanding area of research. The evolution of these endotypes over a very long period of time will be also helpful to determine the trajectory followed by each profile. These researches will bring new knowledge to place strategies for prevention, early diagnosis and treatment of diseases adapted to patients and their risk factors.

In conclusion, all these research opportunities highlight potentially fruitful avenues of integrative approaches and identifications of profiles based on environmental, biological and/or health data. However, it should be point out that no biobank is inexhaustible and said again that study design used to recruit participants, biospecimen sampling schema, and which omics are integrated in the study design will highly influence the results. Large samples but also representativeness, reproducibility or triangulation, and collaboration between biobanks will be crucial.

## Data Availability Statement

The original contributions presented in the study are included in the article/Supplementary Material, further inquiries can be directed to the corresponding author/s.

## Ethics Statement

The studies involving human participants were reviewed and approved by The CONSTANCES Cohort project has obtained the authorization of the National Data Protection Authority on March 3, 2011 (Commission Nationale de l'Informatique et des Libertés—CNIL). CONSTANCES was approved by the National Council for Statistical Information (Conseil National de l'Information Statistique—CNIS), the National Medical Council (Conseil National de l'Ordre des Médecins—CNOM), and the Institutional Review Board of the National Institute for Medical Research-INSERM. The Constances Biobank has received the agreement of the CCP (Comité de protection des personnes du Sud-Est: Committee for the Protection of Persons in the South-East) on 11 April 2018, the authorization from the CNIL (Commission nationale informatique et libertés) on July 19, 2018. The patients/participants provided their written informed consent to participate in this study.

## Author Contributions

JH and RN wrote the manuscript. JH designed the biobank scheme. MG provided sociodemographic and prevalence of major health outcomes data. ES provided project management. DB set up the data processing. KB supervised the biobanking process and revised the description of the biobank. SL, AO, and FR contributed to the monitoring of the inclusion centers. SG contributed to the integration of the traceability data in the CONSTANCES database. MZ approved the manuscript.

## Conflict of Interest

The authors declare that the setting-up of the CONSTANCES Biobank was conducted in the absence of any commercial or financial relationships that could be construed as a potential conflict of interest.

## Abbreviations

ARE: assistant de recherche épidémiologique (Epidemiological Research Assistant)
BIS: Biobanking Information System
COPIL: Comité de pilotage (Steering Committee)
CSBC: CONSTANCES Service Desk
GeT: Greiner Health Technologies solution
HPC: Centres d'examens de santé (Health Prevention Center)
HPE: examen de prévention en santé (Health Prevention examination)
IBBL: Integrated Biobank of Luxembourg
ID: Identifiant
INSERM: Institut national de la recherche médicale (National Institute of Health and Medical Research)
LIMS: Laboratory Management System
PSOP: Personalized Standard Operating Procedure
QMS: Quality Management System.
