# Supplementary material for: The CONSTANCES Cohort Biobank: An Open Tool for Research in Epidemiology and Prevention of Diseases
Source: Front Public Health. 2020 Dec 10;8:605133. doi: 10.3389/fpubh.2020.605133 (PMC7758208; doi:10.3389/fpubh.2020.605133)
Supplement: Supplementary file 1 [file Table_1.DOC]

Supplementary Table S1: CONSTANCES traceability data dictionary

| **Variable List** | **e.g.** |
| --- | --- |
| Sample Collection Data |  |
| Tube barcode | BS2W1A0AA6 |
| Constances ID | 8xxxxx47 |
| Name of operator | 23x9 |
| CES | CES-75x |
| Consent (Yes) |  |
| Fasting for 12h (Yes/No) |  |
| Menstruation (Yes/No) |  |
| Tube type | EDTA |
| Expiry date | 01.mars.19 |
| Expired tube |  |
| Collection date/time | 09.11.2018 08:06 |
| Pre-processing Data |  |
| Centrifugation time | 09.11.2018 08:48 |
| Centrifugation speed | 2000 |
| Centrifugation brake | 9 |
| Centrifugation temperature | 20 |
| Centrifugation duration | 15 |
| Deviation (CES) |  |
| Deviation "centrifugation parameter" |  |
| Deviation "centrifugation impossible" |  |
| Deviation "collection impossible" |  |
| Deviation "damaged tube" |  |
| Deviation "collection refused" |  |
| Deviation "collection tube not inverted" |  |
| Comment "other" |  |
| Deviation "insufficient volume" |  |
| Deviation " damaged tube stored in fridge" |  |
| Deviation "damaged tube after centrifugation" |  |
| Deviation "no temporary storage at 4°C" |  |
| Time of storage at 4°C |  |
| Sample temporary stored at RT | 09.11.2018 09:06 |
| Sample Receipt |  |
| MMUNSCH |  |
| Sample receipt date/time | 10.11.2018 07:32 |
| CES - poolbox origin | CES-7xx |
| Poolbox receipt date/time | 10.11.2018 07:32 |
| Temperature excursion during transport (Yes/No) |  |
| Number of collection forms received | 2 |
| Deviation (collection/receipt) |  |
| Deviation code collection/receipt |  |
| Deviation name collection/receipt |  |
| Comment (operator) |  |
| Deviation origin | CES-3x1 |
| Sample Data |  |
| Identifier | S-180xx2-00xx9 |
| Storage status | Temporary in Lab |
| Destruction reason | Consumed |
| Sample type | PLA |
| Container type | FluidX_2ml_J2D_Ext |
| Storage location | /LN2-11-BIOREP/SA/T121/B/2/SA00314735/164 |
| Storage condition | BoxPos |
| Preparation method identifier | M019 |
| Preparation method name | Plasma Aliquoting |
| Quantity | 450 |
| Unit | µl |
| Parent identifier | SF-140xx8-22xx18 |
| Other tubes from same family | S-190xxx-024x3, S-190xxx-024xx, S-190xxx-024xx, S-190xx-02xx6, S-190xxx-024xx, S-190xxx-002xx |
| Treatment type(s) | Automatic_Processing, Centrifugation, Aliquoting |
| Alias |  |
| Alias ID | 7xxxxxx0, CN2W1A09D3, FD06xx0564 |
| Audit trail |  |
| Deviation (samples) |  |
| Deviation date | 26-JUN-2015 16:38:26 |
| Distribution |  |
| Sample centrifugation |  |
| Centrifugation temperature | RT |
| Centrifugation speed | 2000 |
| Centrifugation duration | 20 |
| Centrifugation acceleration | 7 |
| Centrifugation brake | 5 |
| Centrifugation program | 2000g - 20min - 7acc - 5br - 4°C |
| Centrifugation date/time | 13-APR-2017 15:51:00 |
| Centrifugation comment(s) | time updated |
| Parent Data |  |
| Parent Identifier | S-18xxxx-00xx7 |
| Parent storage status | In Circulation |
| Parent destruction reason |  |
| Parent sample type | Blood |
| Parent container type | EDTA_9ml |
| Children Data |  |
| Child storage status | In Circulation |
| Child destruction reason |  |
| Child sample type | BUF |
| Child container type | FluidX_0_7ml_J2D_Ext |
| Number of children | 7 |
| Nomber of PLA children | 5 |
| Test Data |  |
| Test approval status | Approved |
| Intrument code | COBAS-01 |
| Assay protocol code | M091 |
| Parameter |  |
| Parameter tested | Icterus abs |
| Parameter value | 0.0114 |
| Parameter unit | abs |
